# Anesthetic Management of Unanticipated Subglottic Stenosis in a Patient Undergoing Coronary Artery Bypass Graft Surgery

**DOI:** 10.7759/cureus.56110

**Published:** 2024-03-13

**Authors:** Claudia Wei, Dominique Wreh, Jacy Gressen, Anvinh Nguyen

**Affiliations:** 1 Anesthesiology, Baylor College of Medicine, Houston, USA

**Keywords:** left main coronary artery disease (lmcad), prolonged intubation, difficult airway management, airway dilation, subglottic stenosis

## Abstract

A 62-year-old female presented for a scheduled coronary artery bypass graft (CABG) and was found to have an unexpected subglottic stenosis during routine intubation. The case was aborted and six days later, the patient underwent lasering of the stenotic subglottic region and airway balloon dilation. In this case report, causes of subglottic stenosis and surgical/anesthetic management of the condition are discussed. The management of subglottic stenosis in this patient was complicated by concurrent severe coronary artery disease (CAD) involving the left main coronary artery and timing of airway surgery relative to interventions for her CAD. In situations of undiagnosed subglottic stenosis, anesthesiologists should be familiar with airway management based on the location and severity of the stenosis. Close multidisciplinary team management is required for patients who have other complex comorbidities.

## Introduction

Subglottic stenosis is described as “the narrowing of the upper airway, which lies between the vocal folds and the lower border of the cricoid cartilage” [1]. Causes of subglottic stenosis include congenital, acquired, and idiopathic, with acquired subglottic stenosis being the most common. The most common etiology of acquired stenosis is trauma such as from prolonged endotracheal intubation [1-3]. Other causes include infection, inflammation, radiation exposure, and burns [1]. Stridor is usually the primary presenting symptom. In patients with a known history of prolonged intubation, a careful preoperative history and physical and chart review should be performed to look for signs or symptoms of subglottic stenosis to avoid unexpected airway complications intraoperatively. Anesthesiologists should be familiar with airway management for subglottic stenosis of varying levels. 

## Case presentation

A 62-year-old female with a past medical history of coronary artery disease (CAD) requiring three drug-eluting stents, congestive heart failure with reduced ejection fraction (EF 30-34%), chronic obstructive pulmonary disease (COPD), poorly controlled diabetes, and paroxysmal atrial fibrillation presented for a planned CABG for severe multi-vessel CAD including 60% stenosis of the left main coronary artery. Preoperatively, the patient endorsed mild chronic dyspnea with exertion but otherwise denied having chest pain, worsening shortness of breath, nausea, vomiting, and acid reflux. She had a previous hip replacement surgery under general anesthesia but denied any complications. The preoperative CT chest report did not note any airway abnormalities. A preop pulmonary function test (PFT) performed demonstrated “suboptimal flow volume loops” but the report stated, “normal spirometry, mildly reduced (diffusing capacity for carbon monoxide, forced expiratory volume in 1 second, and forced vital capacity) similar to compared to prior.” Her preoperative cardiopulmonary physical exam was unremarkable.

She was brought into the operating room with a pre-induction arterial line; two 18 gauge peripheral intravenous lines and standard monitors were placed. Induction was uneventful and bag-mask ventilation (BMV) was successful after paralytics were administered. A grade 1 view was achieved with a Macintosh 3 blade and a 7.0 endotracheal tube (ETT) was advanced past the cords but could not be fully inserted due to resistance. A repeat attempt was made with a 6.0 ETT but again resistance was noted when attempting to fully advance the ETT. She was returned to BMV between attempts. A laryngeal mask airway (LMA) was then placed, and the patient was successfully mechanically ventilated. A fiberoptic examination of the airway then revealed significant subglottic stenosis and the otolaryngology (ENT) team was consulted intraoperatively. Airway balloon dilation was initially considered to facilitate proceeding with the CABG. However, after extensive discussion between the ENT, cardiothoracic surgery, and anesthesia teams, dilation was ultimately not performed. All teams felt the concern for significant airway bleeding with systemic heparinization for cardiopulmonary bypass outweighed proceeding with an elective CABG for a patient who had been stable on the floor preoperatively. The patient subsequently emerged from anesthesia and was extubated to supplemental oxygen via a non-rebreather mask. After extubation, the patient’s oxygen saturation remained stable, no stridor was noted, and the patient denied any dyspnea. She was transferred to the ICU awake and in stable condition.

Postoperatively, when discussing the situation with the patient, a family member recalled prolonged intubation secondary to an episode of diabetic ketoacidosis (DKA). As her respiratory status remained stable, she was transferred to the floor the next day. After further discussions regarding surgical risk between the cardiology and cardiothoracic surgery teams, the decision was made to have cardiology perform percutaneous coronary intervention (PCI) in the subsequent days and to follow up with ENT outpatient. However, on postoperative day 3, the patient began to develop mild dyspnea and stridor with exertion. After a multidisciplinary discussion, the decision was made to perform airway dilation prior to PCI.

**Figure 1:**
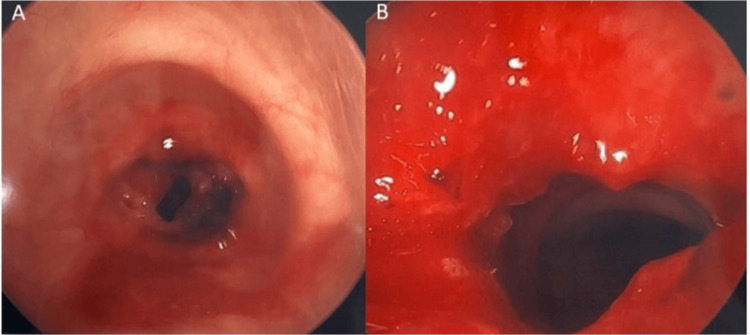
Panel A demonstrates the patient’s stenotic subglottic region prior to dilation; Panel B demonstrates the subglottic region post-dilation with significant improvement in airway diameter

Six days after her initial scheduled CABG, the patient underwent airway dilation surgery with ENT. A CO_2_ laser was used to make cuts along the scar bands of the stenosis. Balloon dilation was performed and Kenalog was injected to reduce recurrent scar formation. Significant improvement in the tracheal stenosis was observed. Figure 1 demonstrates the patient’s trachea before and after dilation. The patient emerged uneventfully and recovered well in the post-anesthesia care unit. Two days after the airway dilation, the patient underwent PCI of the left main coronary artery and was discharged the next day in stable condition.

## Discussion

Subglottic stenosis has various etiologies, with each associated with widely ranging incidence rates. Idiopathic subglottic stenosis is categorized as a rare disease with an estimated annual incidence of 0-2% while subglottic stenosis as an iatrogenic consequence of laryngotracheal intubation is estimated to be 6-21% [1]. Pressure from an endotracheal tube can lead to pressure necrosis, which causes edema and ulceration of the mucosa in the subglottic region. Inflammatory changes lead to the formation of granulation tissue, which leads to airway stenosis [1]. Symptoms of subglottic stenosis include stridor, aphonia, hoarseness, dyspnea, and retractions depending on severity. A thorough patient history can help identify risk factors for subglottic stenosis such as prolonged intubations, autoimmune disorders, and gastroesophageal reflux disease [2-5]. In our case, we suspected the patient’s intubation during her DKA episode was likely the inciting factor for her subglottic stenosis.

In patients with symptoms suspicious for subglottic stenosis, various diagnostic tests can be done, including computed tomography imaging and PFTs. However, the gold standard for confirming the diagnosis is via flexible fiberoptic examination of the airway. Table 1 below demonstrates two systems for the classification of stenosis severity [6,7]. 

**Table 1:** Two different classification systems for the severity of subglottic stenosis Source: [6,7]

Classification System	Severity
Cotton-Myer Classification	Grade I – 0-50% obstruction
	Grade II – 51 to 70% obstruction
	Grade III – 70-99% obstruction
	Grade IV – No detectable lumen
Freitag Classification	Grade 0 – 0% stenosis
	Grade 1 – 25% stenosis
	Grade 2 – 50% stenosis
	Grade 3 – 75% stenosis
	Grade 4 – 90% stenosis
	Grade 5 – No detectable lumen

Management of subglottic stenosis depends on the level of severity. Asymptomatic patients generally can be observed. Treatment is generally reserved for patients with severe stenosis (> 75%) or patients who have had related complications [8]. Symptomatic patients generally receive various treatments, including balloon dilation, injection therapy, stent placement, or ablation with lasers or cryotherapy. The choice of therapy is at the discretion of the managing physician and depends on the severity of the stenosis. In the most severe cases, open tracheal surgery or a tracheostomy might be necessary [8].

With unanticipated subglottic stenosis, the anesthesia team must make rapid decisions. In cases where the stenotic region is more distal, an ETT can potentially be advanced to have the cuff through the cords and emergency surgery can be performed if needed. If the lesion is more proximal to the glottis, the anesthesia team would have to weigh the urgency of the surgery versus the risks of performing surgery with ventilation via BMV, LMA, or even jet ventilation and no secure airway [9]. In cases of unanticipated subglottic stenosis and difficulty with intubation, anesthesia team members should always refer to the difficult airway algorithm. For our patient, after two attempts of unsuccessful advancement of the ETT, we returned to BMV and then inserted an LMA. Had we not been able to BMV or ventilate through the LMA, the next step would have been an invasive airway [10].

In our case, the proximity of the stenotic region to the glottis prevented us from passing the cuff of the ETT fully past the glottic opening. We discussed potentially intubating with a smaller ETT for the CABG procedure but elected not to rely on a small ETT for prolonged postoperative ventilation after critical surgery. Fiberoptic bronchoscopy could not be effectively performed via a small ETT as well. Furthermore, intubation could lead to worsening edema in the stenotic region and the inability to safely extubate our patient. This could ultimately lead to our patient requiring a tracheostomy. Immediate airway dilation was suggested but not performed due to concern for airway bleeding from full systemic heparinization needed for CABG and the risk of continuing coagulopathy after cardiac surgery [11].

Ultimately, because the CABG was elective and the patient was stable from a cardiopulmonary perspective, we decided these risks that were not previously discussed with the patient outweighed the benefits. In situations where the CABG had been for an emergent indication, we likely would have discussed the risks and benefits with the patient’s family member prior to proceeding with an airway intervention.

A multidisciplinary discussion was also held regarding the timing of the patient’s airway dilation relative to her PCI. Because the PCI would involve a very high-risk lesion in the left main coronary artery, which could lead to hemodynamic instability requiring emergent intubation, all teams felt that it would be better to stabilize the patient’s airway prior to her PCI.

## Conclusions

Subglottic stenosis is a rare disease but can be caused by prolonged intubation. In this case report, we discussed the management of a patient found to have incidental subglottic stenosis during intubation for a CABG. Although she did not have apparent risk factors for subglottic stenosis preoperatively, further prompting postoperatively revealed a history of prolonged intubation secondary to a DKA episode. Previously undiagnosed subglottic stenosis poses airway challenges to the anesthesia team and anesthesiologists should be familiar with airway management for subglottic stenosis of varying levels. This case highlights the importance of multidisciplinary discussion in managing a patient with an incidental finding of subglottic stenosis with concurrent complex cardiac comorbidities.
